# Clinical judgment performance of undergraduate Nursing students*

**DOI:** 10.1590/1518-8345.4843.3452

**Published:** 2021-06-28

**Authors:** Vanessa Brito do Canto, Tatianne Gonçalves da Silva, Gutembergue Aragão dos Santos, Emilia Campos de Carvalho, Sheila Coelho Ramalho Vasconcelos Morais, Cecília Maria Farias de Queiroz Frazão

**Affiliations:** 1 Universidade Federal de Pernambuco, Departamento de Enfermagem, Recife, PE, Brazil.; 3 Universidade de São Paulo, Escola de Enfermagem de Ribeirão Preto, PAHO/WHO Collaborating Centre for Nursing Research Development, Ribeirão Preto, SP, Brazil.; 4 Universidade Federal de Pernambuco, Recife, PE, Brazil.

**Keywords:** Nursing Education, Nursing Students, Clinical Decision-Making, Clinical Competence, Nursing Process, Self-Assessment, Educação em Enfermagem, Estudantes de Enfermagem, Tomada de Decisão Clínica, Competência Clínica, Processo de Enfermagem, Autoavaliação, Educación en Enfermería, Estudiantes de Enfermería, Toma de Decisiones Clínicas, Competencia Clínica, Proceso de Enfermería, Autoevaluacíon

## Abstract

**Objective:**

to evaluate the reported performance regarding clinical judgment by undergraduate Nursing students.

**Method:**

a cross-sectional study with the application of the *Lasater Clinical Judgment Rubric-Brazilian Version* in 166 undergraduate Nursing students from a Brazilian public university. The data were analyzed descriptively and analytically (by comparing the level of clinical judgment among students from the initial, intermediate, and concluding groups). The following tests were applied: Chi-square, Fisher’s Exact and Kruskal-Wallis, and a p-value of 0.05 was adopted. The reliability of the global instrument (Cronbach’s alpha) was 0.786.

**Results:**

of the 166 students, 65.7% evaluated themselves as proficient in relation to the reported performance on clinical judgment. Of the rubric’s 11 dimensions (focused observation, recognizing deviations from expected patterns, information seeking, prioritizing data, making sense of data, calm and confident manner, clear communication, well-planned intervention/flexibility, being skillful, evaluation/self-analysis, and commitment to improvement), only four groups did not present significant differences among them (p<0.05): focused observation, information seeking, prioritizing data, and calm and confident manner.

**Conclusion:**

the performance on clinical judgment reported as proficient was pointed out by 65.7% of the students and a significant statistical difference was verified in seven dimensions, among beginners, intermediate, and concluding students, compatible with the evolution of learning.

## Abstract (pt)

**Objetivo:**

avaliar o desempenho referido sobre julgamento clínico por estudantes de graduação em enfermagem.

**Método:**

estudo transversal, com a aplicação da *Lasater Clinical Judgment Rubric-Brazilian Version* em 166 estudantes da graduação em enfermagem de uma universidade pública do Brasil. Os dados foram analisados de forma descritiva e analítica (comparando o nível de julgamento clínico entre estudantes dos grupos iniciante, intermediário e concluinte). Aplicaram-se os testes: Qui-quadrado, Exato de Fisher e Kruskal-Wallis, e adotou-se p-valor 0,05. A confiabilidade do instrumento global (alpha de Cronbach) foi de 0,786.

**Resultados:**

dos 166 estudantes, 65,7% se avaliaram como proficientes em relação ao desempenho referido sobre julgamento clínico. Das 11 dimensões da rubrica (observação focada, reconhecimento de desvios dos padrões esperados, busca por informações, priorização dos dados, compreensão dos dados, atuação calma e confiante, comunicação clara, intervenção bem planejada/flexibilidade, habilidade técnica, avaliação/autoanálise e comprometimento com o aperfeiçoamento), apenas quatro não apresentaram diferenças significativas entre os grupos (p<0,05): observação focada, busca por informações, priorização dos dados, atuação calma e confiante. Conclusão: O desempenho referido sobre julgamento clínico como proficiente foi apontado por 65,7% estudantes e foi verificada diferença estatística significante, em sete dimensões, entre os iniciantes, intermediários e concluintes, compatível com a evolução da aprendizagem.

## Abstract (es)

**Objetivo:**

evaluar la capacidad referida de juicio clínico de estudiantes de licenciatura en enfermería.

**Método:**

estudio transversal con aplicación de la Lasater *Clinical Judgment Rubric-Brazilian Version* en 166 estudiantes de licenciatura en enfermería de una universidad pública de Brasil. Los datos se analizaron de forma descriptiva y analítica (comparando el nivel de juicio clínico entre los estudiantes de los grupos principiante, intermedio y avanzado). Se aplicaron las pruebas: Chi-cuadrado, Exacta de Fisher y Kruskal-Wallis, y se adoptó un p-valor de 0,05. La confiabilidad del instrumento global (alfa de Cronbach) fue de 0,786.

**Resultados:**

de los 166 estudiantes, el 65,7% se evaluó como competente en relación con la capacidad referida de juicio clínico. De las 11 dimensiones de la rúbrica (observación enfocada, reconocimiento de desviaciones de los estándares esperados, búsqueda de información, priorización de datos, comprensión de datos, desempeño tranquilo y seguro, comunicación clara, intervención bien planificada/flexibilidad, habilidad técnica, evaluación/autoanálisis y compromiso con la mejora), solo cuatro no mostraron diferencias significativas entre grupos (p<0,05): observación enfocada, búsqueda de información, priorización de datos, desempeño tranquilo y seguro.

**Conclusión:**

el 65,7% de los estudiantes consideró que poseía juicio clínico competente y hubo una diferencia estadísticamente significativa, en siete dimensiones, entre principiantes, intermedios y avanzados, compatible con la evolución del aprendizaje.

## Introduction

One of the difficulties in the education of future health professionals is the development of the reasoning process related to clinical judgment for decision-making. To implement their work method, nurses need to think and develop skills that enable problem-solving by means of effective clinical judgment and, consequently, efficient decision-making^(^ 1 ^)^.

Clinical judgment comprises a conclusion about problems or needs of the individuals, with consequent decision-making about the situation, modifying approaches as necessary, according to the patient’s responses^(^ 2 ^)^.

It consists of four stages, namely: noticing, interpreting, responding, and reflecting. Initially, the professional identifies and evaluates the clinical situation of the individual, and this process suffers direct interferences of the perception and discernment capability of the nurse, of their relationship with the client and with the health service. Subsequently, the nurse interprets the data by means of reasoning patterns and determines conducts appropriate to the case. Finally, they reflect on the results of the established actions and about their performance during the entire process^(^ 2 ^)^.

Clinical judgment in the nurses’ professional practice is essential for decision-making. Thus, the process to acquire this skill must primarily occur in the initial stages, that is, still during undergraduation. It is up to the Nursing schools to provide the students with integration of theory and practice with a focus on improving clinical judgment^(^ 3 ^-^ 4 ^)^.

The development of knowledge and the acquisition of experiences allow establishing assertive decision-making in a safe manner. Thus, it is relevant to consider other teaching methods, in addition to the conventional ones, such as discussion patients’ cases in high-fidelity clinical simulations^(^ 4 ^-^ 6 ^)^.

Therefore, it is necessary for the educators to implement techniques that help to obtain clinical judgment by the students, as well as their evaluation, in order to eliminate avoidable harms to the patients, aiming at their safety^(^ 7 ^)^.

In this perspective, based in the four stages of clinical judgment^(^ 2 ^)^: Noticing, Interpreting, Responding, and Reflecting, a rubric was developed that evaluates the performance of clinical judgment, called Lasater Clinical Judgment Rubric (LCJR).

The LCJR helps in the teaching-learning process to the extent that it is useful to identify the gaps in the development of skills and attitudes, where teachers can intervene as they inform the students regarding their performance^(^ 8 ^)^. This allows the students to evaluate the development of clinical judgment and their own progress, identifying the areas that need to be improved to be successful^(^ 9 ^)^.

This rubric was adapted to the Brazilian culture and semantics in 2016, receiving the name of Lasater Clinical Judgment Rubric - Brazilian Version (LCJR-BV)^(^ 6 ^)^. The LCJR-BV can be associated with the teaching-learning methodologies as a resource for training evaluation, whether by the intermediation of the teachers or in the format of self-evaluation by the students themselves, as well as a tool with a focus on the identification of the limitations and the provision of feedback about the items that should be improved.

Thus, ratifying the importance of using the LCJR-BV, this study had as objective to evaluate the reported performance on clinical judgment by undergraduate Nursing students.

## Method

This is a quantitative, descriptive, and cross-sectional study. It was conducted during the second semester of 2019 in a public university located in Recife, Pernambuco, Brazil.

The Nursing department of this university comprises ten terms of the undergraduate course, in the daytime shift. In the third term, there is a subject which addresses the theme of clinical judgment to be applied in the next terms, when the students begin their practical experiences in the routines of the health services.

The population was composed of 183 students enrolled in that same year, from the fourth to the tenth term in the Nursing undergraduate course. To recruit the sample, the convenience sampling strategy was used, adopting the following inclusion criteria: being duly enrolled in the undergraduate Nursing course, being 18 years old or older, and having passed the subject involving the theme in question: clinical judgment.

The sample total was 166 students who accepted to participate in the research and met the eligibility criteria. 17 students did not participate in the research due to: refusal (reason not informed) and absence from the classroom during the period that followed data collection.

The following instruments were self-applied: questionnaire for sociodemographic characterization and the LCJR-BV rubric, as well as the signed Free and Informed Consent Form – FICF. The sociodemographic instrument investigated the following variables: gender, age, undergraduation period, complementary training (another undergraduate course and/or Nursing technician/assistant course), and experience in the professional area.

The Brazilian version of the LCJR used in this research presents 11 dimensions: focused observation, recognizing deviations from expected patterns, information seeking, prioritizing data, making sense of data, calm and confident manner, clear communication, well-planned intervention/flexibility, being skillful, evaluation/self-analysis, and commitment to improvement. Such dimensions explicit developmental descriptors, enabling classification in four levels: beginning – 1 point, developing – 2 points, accomplished – 3 points, and exemplary – 4 points. The final score ranges from 11 to 44, and the best capability for clinical judgment is attributed to the highest scores^(^ 6 ^)^.

In 2018, the LCJR-BV was evaluated regarding its psychometric properties (discriminant validity, reliability, and dimensionality), with a reliability result to evaluate the development of clinical judgment in the Nursing student. This result was obtained by internal consistency analysis (Cronbach’s alpha of 0.889)^(^ 10 ^)^ and, in the present study, with 0.786 for the total value of the instrument.

For each domain there is a Likert type scale, in which the score varies from 1 to 4, corresponding to the “exemplary”, “accomplished”, “developing”, and “beginning” levels. These were respectively replaced by the descriptions “A”, “B”, “C”, and “D” in the applied instrument, in order not to induce the choice of a certain level by the participants.

Data collection took place during August and September 2019. It was carried out after inviting the participants and after duly clarifying the research objective, instruments to be used (demographic questionnaire, and the LCJR-BV), data confidentiality, and signature of the Free and Informed Consent Form – FICF. The instruments were delivered to the participants before the in-person classes began. The students should return them filled in after a maximum of 40 minutes.

The data from the research instruments were typed and compiled by double entry in the SPSS software, version 25.0. The participants’ data made up three groups, namely: beginners, those enrolled in the classes of the fourth and fifth term, intermediate, from sixth to eighth term, and concluding, from ninth and tenth term. For the descriptive analysis, the data were presented in absolute and relative frequency. In the inferential analysis, the Chi-square for homogeneity, Fisher’s Exact, and Kruskal-Wallis tests were used, adopting a significance level of 5%.

The research was conducted after approval by the institution’s Research Ethics Committee, under CAAE protocol 12783119.6.0000.5208 and Opinion number: 3,436,993, according to the ethical-legal aspects supported by Resolution No. 466/2012^(^ 11 ^)^.

## Results

Of the 166 (100%) students, 147 (88.6%) were female; with a mean age of 22.4 years old (minimum of 18; maximum of 46); the group of beginners was composed of 51 students, while there were 79 in the intermediate group, and 36 participants in the concluding group.

Regarding complementary training, eight students (4.2%) mentioned having another graduation course, among which the following were cited: bachelor’s degree in home economics, biological sciences, tourism, social work, administration, and an unspecified one. In addition, 9 (5.4%) had the Nursing technician course and, among these, 4 (2.4%) currently work in the area (minimum of 3 years; maximum of 5 years), according to Table 1.

**Table 1:** Numerical and percentage distribution of the Nursing students according to the sociodemographic variables, complementary training, and current function. Recife, PE, Brazil, 2020

Variables		n	%
Gender	Female	147	88.6
	Male	19	11.4
Term	Fourth	24	14.5
	Fifth	27	16.3
	Sixth	25	15.1
	Seventh	27	16.3
	Eighth	27	16.3
	Ninth	18	10.8
	Tenth	18	10.8
Other Higher Education course	No	158	95.2
	Yes	8	4.8
Professionalizing course	No	157	94.6
	Technician	9	5.4
	Assistant	0	0.0
Works as a technician	No	162	97.6
	Yes	4	2.4

The self-evaluation on clinical judgment by the LCJR-BV showed, from the total score, that no student was classified with the “beginning” performance, 15 were “developing”, 109 were “accomplished” and 42 were “exemplary”. In addition to that, the “accomplished” classification prevailed in all the terms, as verified in Table 2.

**Table 2:** Frequency of the classification of the development levels by the total score of the LCJR-BV* by terms of the Nursing course. Recife, PE, Brazil, 2020

Terms		Classification		
		Developing	Accomplished	Exemplary
4th (n=24)	Beginner	3 (12.5%)	19 (79.2%)	2 (8.3%)
5th (n=27)		5 (18.5%)	15 (55.6%)	7 (25.9%)
6th (n=25)	Intermediate	2 (8%)	17 (68%)	6 (24%)
7th (n=27)		2 (7.4%)	13 (48.1%)	12 (44.4%)
8th (n=27)		2 (7.4%)	21 (77.8%)	4 (14.8)
9th (n=18)	Concluding	0	13 (72.2%)	5 (27.8%)
10th (n=18)		1 (5.6%)	11 (61.1%)	6 (33.3%)
Total (n=166)		15 (9.0)	109 (65.7)	42 (25.3)

* Lasater Clinical Judgment Rubric - Brazilian Version

In relation to the groups (beginners - fourth and fifth terms, intermediate - sixth to eighth terms, and concluding - ninth and tenth terms), in the beginner, 15.7% were classified as “developing”, 66.7% as “accomplished”, and 17.6% as “exemplary”. In the intermediate group, 7.6% were “developing”, 64.6% were “accomplished” and 27.8%, “exemplary”. And among the concluding students, 2.8% fit in the “developing” category, 66.7% in “accomplished”, and 30.5% in “exemplary”.

The analysis of the LCJR-BV dimensions with the categories self-assessed by the students observed that there was a significant statistical association in the total score and in seven dimensions (p<0.05) between the groups: Recognizing deviations from expected patterns; Making sense of data; Communication; Intervention; Being skillful; Evaluation; and Commitment (Table 3).

**Table 3:** Distribution of the scores of the LCJR-BV* dimensions, according to the Nursing students grouped in beginners, intermediate, and concluding. Recife, PE, Brazil, 2020

Evaluated domain	Levels	Study period			p-value
		Beginner 4th and 5th	Intermediate 6th to 8th	Concluding 9th and 10th	
Focused Observation	Exemplary	1 (1.9%)	1 (1.3%)	0 (0.0%)	0.696^‡^
	Accomplished	11 (21.6%)	14 (17.7%)	3 (8.3%)	
	Developing	24 (47.1%)	38 (48.1%)	21 (58.3%)	
	Beginning	15 (29.4%)	26 (32.9%)	12 (33.3%)	
Recognizing deviations from expected patterns	Exemplary	3 (5.9%)	2 (2.5%)	0 (0.0%)	0.003^‡^
	Accomplished	24 (47.1%)	22 (27.8%)	4 (11.1%)	
	Developing	21 (41.1%)	49 (62.1%)	26 (72.2%)	
	Beginning	3 (5.9%)	6 (7.6%)	6 (16.7%)	
Information seeking	Exemplary	2 (3.9%)	1 (1.3%)	0 (0.0%)	0.222^‡^
	Accomplished	5 (9.8%)	5 (6.3%)	0 (0.0%)	
	Developing	23 (45.1%)	28 (35.4%)	16 (44.4%)	
	Beginning	21 (41.2%)	45 (57.0%)	20 (55.6%)	
Prioritizing data	Exemplary	1 (1.9%)	2 (2.5%)	0 (0.0%)	0.886^‡^
	Accomplished	10 (19.6%)	17 (21.5%)	10 (27.8%)	
	Developing	26 (51.0%)	44 (55.7%)	17 (47.2%)	
	Beginning	14 (27.5%)	16 (20.3%)	9 (25.0%)	
Making sense of data	Exemplary	1 (1.9%)	0 (0.0%)	0 (0.0%)	0.005^‡^
	Accomplished	20 (39.2%)	12 (15.2%)	4 (11.1%)	
	Developing	27 (53.0%)	59 (74.7%)	26 (72.2%)	
	Beginning	3 (5.9%)	8 (10.1%)	6 (16.7%)	
Calm and confident manner	Accomplished	10 (19.6%)	14 (17.7%)	7 (19.4%)	0.883^†^
	Developing	23 (45.1%)	43 (54.5%)	18 (50.0%)	
	Beginning	18 (35.3%)	22 (27.8%)	11 (30.6%)	
Clear communication	Exemplary	1 (1.9%)	0 (0.0%)	1 (2.7%)	0.015‡
	Accomplished	5 (9.8%)	9 (11.4%)	2 (5.6%)	
	Developing	30 (58.9%)	31 (39.2%)	10 (27.8%)	
	Beginning	15 (29.4%)	39 (49.4%)	23 (63.9%)	
Well-planned intervention/flexibility	Exemplary	3 (5.9%)	0 (0.0%)	0 (0.0%)	0.029^‡^
	Accomplished	12 (23.5%)	7 (8.8%)	3 (8.3%)	
	Developing	9 (17.6%)	16 (20.3%)	5 (13.9%)	
	Beginning	27 (53.0%)	56 (70.9%)	28 (77.8%)	
Being skillful	Exemplary	2 (3.9%)	0 (0.0%)	0 (0.0%)	0.002^‡^
	Accomplished	12 (23.5%)	4 (5.0%)	1 (2.8%)	
	Developing	33 (64.8%)	65 (82.3%)	27 (75.0%)	
	Beginning	4 (7.8%)	10 (12.7%)	8 (22.2%)	
Evaluation/self-analysis	Exemplary	0 (0.0%)	1 (1.3%)	0 (0.0%)	0.013^‡^
	Accomplished	12 (23.5%)	7 (8.8%)	2 (5.6%)	
	Developing	27 (53.0%)	52 (65.8%)	31 (86.1%)	
	Beginning	12 (23.5%)	19 (24.1%)	3 (8.3%)	
Commitment to improvement	Accomplished	7 (8.8%)	2 (5.6%)	2 (5.6%)	0.030^‡^
	Developing	31 (60.8%)	41 (51.9%)	17 (47.2%)	
	Beginning	13 (25.5%)	36 (45.6%)	17 (47.2%)	
Total Score	-	32.0 [6.0]	35.0 [4.0]	36.0[4.0]	0.001^§^

* Lasater Clinical Judgment Rubric - Brazilian Version;

† p-value of the Chi-square test for homogeneity;

‡ p-value of the Fisher's Exact Test Value

§ p-value of the Kruskal-Wallis test

When comparing the groups, two by two, there was significance between the groups of students from the 4th to 5^th^ and 6^th^ to the 8^th^ term (p-value = 0.003) and for the comparison of the students from 4th to 5^th^ and 9^th^ to 10^th^ term (p-value = 0.001), the group from 4^th^ to 5^th^ term being the one that presented the lowest median (32.0 points) of the total score in comparison to the group from 6th to the 8^th^ term (35 points) and 9^th^ to 10^th^ term (36.0 points). And in the comparison of the 6^th^ to 8^th^ term group with the 9th to 10^th^ term group, there was no significant difference (p-value = 0.304), indicating that the distribution of the evaluation score of the two groups is similar.

## Discussion

To perform Nursing care in a safe and accurate manner, it is essential to acquire and develop cognitive and behavioral skills, which are necessary for the construction of clinical judgment for decision-making during the Nursing process^(^ 12 ^)^.

In addition, faced with challenging, complex, and unpredictable health need demands, in the current times, the students in the Nursing undergraduate course must be trained to be nurses capable of thinking critically, demonstrate proper clinical reasoning skills and excellent clinical judgment in real situations of patient care^(^ 13 ^)^.

To do so, it is necessary to offer high-quality education in Nursing in which the professors implement safe techniques that help in the acquisition process of clinical judgment by the students, as well as instruments that allow for the evaluation of these processes in order to provide feedback to the teacher. The LCJR has shown to be a safe instrument for such purpose according to a study^(^ 14 ^)^, as well as the results of this research that enabled to distinguish the clinical judgment of students with different experiences.

With the application of the LCJR-BV in this research, it was noticed that the groups (beginner, intermediate and concluding) obtained high total scores and that, of the 11 dimensions evaluated in LCJR-BV between the groups, seven presented a significant difference: Recognizing deviations from expected patterns; Making sense of data; Communication; Intervention; Being skillful; Evaluation; and Commitment.

The beginner group (4^th^ and 5^th^ terms) obtained the lowest median (32.0 points) of the total score compared to the intermediate group (6^th^ to 8^th^ terms) and concluding group (9^th^ to 10^th^ terms). The aforementioned dimensions are part of a cross-sectional teaching-learning process, making the student advance gradually in each term in the cognitive and technical skills.

Clinical judgment is related to the previous practical experiences of each individual^(^ 8 ^)^, a fact that was observed in a research study carried out with experienced and beginner nurses subjected to clinical simulation and scoring by means of the LCJR rubric, which pointed out a significant difference between the results of the groups^(^ 15 ^)^. This was also evidenced in the reliability and validity research of the LCJR-BV, in which all dimensions obtained significant differences between the groups of beginners and advanced students in the Nursing course.

In line with the fact that practical experimentation significantly influences the clinical judgment ability, in other research studies and through an evaluation by the rubric in question, the acquisition of this skill among groups that live more experiences than others could be observed. For example, in a Chinese study, students subjected to more practical experiences, through simulation teaching, obtained greater performance in all the subdomains of the LCJR Chinese version, in relation to those who were exposed only to traditional learning methodologies^(^ 7 ^)^. Intervention sessions with clinical simulation and, later, discussion moments supported by a model of clinical judgment, self-evaluations, and observer evaluations by means of the LCJR, evidenced an improvement trend of clinical judgment in the neonatal intensive care units, in the United States^(^ 16 ^)^.

In parallel to the application of the LCJR in several cultural contexts in the international and national scene, as well as in the target population (students, professors, nurses in practice) a discussion emerges about the results from different ways of applying the evaluation of the instrument.

In a study carried out in Holland, they obtained the comparison between the rubric’s scores performed by preceptor nurses, by professors, and by the self-evaluations of the students about their hospital practices. Although the differences among the evaluators do not show to be significant, the students were observed to assign higher values, while the professors demonstrated a greater variety of grades^(^ 17 ^)^.

In a similar study, the performance of clinical judgment was investigated from a simulation, both by the analysis of an evaluator and by the students’ self-evaluation. It was verified that self-evaluation reached a higher mean score than the one assigned by the evaluator and, thus, the overconfidence based on presumptuous self-evaluation is harmful to inexperienced nurses and can result in inconsistent patient care^(^ 18 ^)^.

The self-reflexive method, employed when self-evaluation is conducted, involves a complex action, and there may be occasional underestimation or overestimation of the values pointed out by the students^(^ 19 ^)^.

In addition, the importance of using the LCJR-BV as an evaluation instrument is ratified to help the professors during the acquisition process of clinical judgment by the students, as a source of safe feedback to improve or modify implemented teaching strategies.

Furthermore, this study provided an overview of the self-perception of graduation students from different academic levels about clinical judgment, contributing so that the higher education institution reflects about its evaluative methodologies, as a source of improving Nursing education. Therefore, the importance of using the rubric in this context is confirmed. However, in view of the findings of this research, its application only through the students’ self-evaluation method is questioned since they are in concomitant development of other skills, such as criticality and self-reflection on their practices.

The limitations of this study are the type of sampling used (for convenience), not having had all the students enrolled, and the application of the rubric in a punctual manner and not associated with teaching-learning methodologies.

## Conclusion

Through this research, it is possible to verify the level of development of clinical judgment from the point of view of the students, through the LCJR rubric in its Brazilian version. It was observed that the performance of the researched students, in relation to the total score, was mostly framed in the “accomplished” level of the instrument, and that no student fell into the beginner category, even in classes at the beginning of the course. In addition, a significant difference was shown between the beginner, intermediate, and concluding groups in seven of 11 dimensions of the rubric, revealing that such dimensions are part of a cross-sectional teaching-learning process, making the student advance gradually in every term.

Therefore, the relevance of implementing evaluations that encourage the students’ critical reflection on the practice is reinforced, considering that the knowledge pertinent to the nurse’s professional knowledge includes the improvement of cognitive and technical skills.

Consequently, the results of this research ratify the importance of stimulating innovation in teaching, by using evaluative methods in association with those traditionally used, for a qualified training of human resources in health.

The relevance for the elaboration of new research studies with the application of the LCJR-BV is highlighted, as a way to evaluate the teaching methodologies and the process to acquire clinical judgment of undergraduate and graduate students, as well as its application by all those involved in the context (professor, student, and observer).
